# The Prince of Wales's Hospital Fund

**Published:** 1899-01-14

**Authors:** 


### The Institutional Workshop.

### THE PRINCE OF WALES'S HOSPITAL FUND.

### SOME CRITICISMS ANSWERED.

The list of awards of the Prince of Wales's Hospital Fund which we published last week has given general satisfaction—a fact which is indicated by the small amount of criticism which they have evoked. Such criticism has, however, come from two sources, "A Hospital Secretary," who is so afraid of his own views, or so conscious that they are prejudiced that he has not the courage to sign his name, and an anonymous correspondent in, and the Editor of the *Medical Press and Circular*. We dealt with the criticisms of the former, so far as they were material, in a note in last week's issue. The latter criticisms amount to this: First, that the awards made should have been uniform—that is, as we understand it, that every hospital should have had a *pro ratâ* distribution on some basis either of beds, or patients, or something. From enquiries we have made we gather that this *pro ratâ* distribution did not commend itself to the Council of the Prince of Wales's Fund because the reports of the visitors and the position indicated by an analysis of the figures and the financial condition of particular hospitals, coupled with the work done, showed that any such basis must in fact prove unsatisfactory. It is said that such a basis of uniformity would have prevented the Prince of Wales's Fund from granting £5,000 a year each to Guy's Hospital and to the London Hospital respectively. If a reference be made to the first report of the Distribution Committee of the Prince of Wales's Fund, it will be found that, after much consideration, it was decided that the money of the Fund could not be better spent than in aiding, in proportion to their needs and usefulness, the most important of the hospitals. These were defined to be those having 100 or more beds, a test which necessarily included all the hospitals having medical schools. The existence of a school in connection with a hospital was rightly held to give it a strong claim to support, not only because a medical school in itself confers immense benefit on the community, but also because the public criticism to which the officers of such a hospital are perpetually subjected ensures that the treatment of the patients is conducted in accordance with the best existing knowledge. It was further determined that the allocation of any portions of the Fund to the extremely important object of opening closed wards must be done on the understanding that it will be on a more or less permanent footing. It has thus come to pass that by the operation of this last decision no less than 242 additional hospital beds have been placed permanently at the disposal of the sick poor of London. Such a result could not have been accomplished by any uniform system of distribution, for the award of £5,000 to Guy's Hospital represents rather less than the cost of the actual number of additional beds which have, in consequence of this grant, been equipped, opened, and occupied by patients in wards formerly closed. The grant to the London Hospital, where there were no vacant beds, was made as a means of relieving its committee to a large extent of their financial difficulties, and of placing them in a position to have the whole of their buildings and appointments brought up to date and made adequate to the work which they have to undertake for the dense population in one of the poorest portions of the metropolis from which its patients are drawn. It is satisfactory to know that for the reasons stated these larger awards have been very generally approved.

The *Medical Press*, alluding to the annual grants made to nine general hospitals and three special hospitals, asks: "Are we to understand that in the future the hospitals named therein will be paid large grants before the claims of any other institutions will be considered?" This question was inferentially anticipated and answered by the Distribution Committee in their report of last year, wherein they stated, as already quoted, that the allocation of any portion of the funds to the extremely important objects of opening closed wards must be done on the understanding that it will be on a more or less permanent footing. It would never have done to have induced any hospital to open a closed ward, to equip it and maintain it for a year, and at the end of that year to say: "Now you must either close the ward again or find the money from some other source." It follows that the answer to the question of the *Medical Press* must be: "Yes, it is intended to continue these annual grants so long as the position of the various hospitals show that this aid is necessary to maintain the reopened wards." The *Medical Press* further asks, "Are we to conclude that the list is final, and that in succeeding years, should the Fund continue, no other additions will be made to it?" We cannot, of course, say what view the Prince of Wales's Fund Council may take on this point. It is not dealt with in their report. There is, however, one very obvious answer—*i.e.*, that if the permanent income of the Prince's Fund is increased there can be little doubt that other hospitals of repute, which can demonstrate the pressing needs of a permanent addition to their income in order to continue or increase the amount of good already done, will be added to the present list. All, therefore, who are interested in the hospitals, and all well-wishers to the Prince's Fund, will do their utmost to extend its area of contribution and the volume of income at the disposal of the Council of this Fund.

The *Medical Press* expresses the view that it is a mistake to let any award to a single institution exceed, say, £2,500. It maintains that the object of the Prince's Fund "should be to do the greatest good to the greatest number of the charities concerned." Surely the writer has missed the real object for which the Prince's Fund was established. That object was to do the greatest good to the greatest number of sick and suffering Londoners, not to "the greatest number of charities concerned." The Fund has little to do with individual charities as such, but it has much to do with the provision of adequate hospital accommodation for the poor of London. Hence the plan pursued of subsidising those hospitals where there were vacant wards in order to place more beds at the disposal of the really deserving poor is a precise fulfilment of the original object of the Fund, which would not have been fulfilled had this point been lost sight of in favour of the plan indicated by the *Medical Press*. The same writer falls into an error, too, in regard to the West London Hospital. The visitors, we understand, found that there was much overcrowding in the older portions of the hospital and no adequate bath-room accommodation. This state of affairs was allowed to exist, although a new wing had been built containing 75 beds, not one of which had been equipped or occupied. The Council of the Prince of Wales's Fund attached the condition to their grant of £500 to this hospital, that the overcrowding and deficient bath-room accommodation should be remedied immediately. The readiest way of doing this would be for the committee to equip and open the new wing and to leave the older portions of the hospital unoccupied until they have funds sufficient to remedy the undoubted evils attaching to the defective construction and inadequate accommodation of a hygienic character. Such a condition cannot therefore be properly described as "a satire." It amounts, in fact, to a practical suggestion to bring the hospital up to modern requirements by utilizing the new buildings instead of keeping the patients crowded together under insanitary conditions in the older portions of the existing buildings. We welcome the criticism and inquiries of our contemporary because we are confident that the more closely the grants of the Prince of Wales's Fund are examined and the more completely the conditions attached to certain of these awards are understood, the greater will be the volume of public approval in favour of the decisions arrived at. What the Prince of Wales's Fund now wants is local machinery whereby an adequate number of new subscribers may be induced to give something to the hospitals through the Prince's Fund who have not heretofore contributed at all to the hospitals of London.

